# Intratumoral and peritumoral MRI-based radiomics prediction of histopathological grade in soft tissue sarcomas: a two-center study

**DOI:** 10.1186/s40644-023-00622-2

**Published:** 2023-10-26

**Authors:** Liyuan Zhang, Yang Yang, Ting Wang, Xi Chen, Mingyue Tang, Junnan Deng, Zhen Cai, Wei Cui

**Affiliations:** 1 Department of Plastic Surgery, Sichuan Provincial People’s Hospital, School of Medicine,University of Electronic Science and Technology of China, Chengdu, 610000 People’s Republic of China; 2 Department of Radiology, Hospital of Chengdu University of Traditional Chinese Medicine, Chengdu, 610000 People’s Republic of China; 3 Department of Plastic Surgery, The First People’s Hospital of Yibin, Yibin, 644000 People’s Republic of China; 4 Sichuan College of Traditional Chinese Medicine, Mianyang, 621000 People’s Republic of China

**Keywords:** Magnetic resonance imaging, Radiomics analysis, Sarcomas

## Abstract

**Objectives:**

This study aims to develop a model based on intratumoral and peritumoral radiomics from fat-suppressed T2-weighted(FS-T2WI) images to predict the histopathological grade of soft tissue sarcoma (STS).

**Methods:**

This retrospective study included 160 patients with STS from two centers, of which 82 were low-grade and 78were high-grade. Radiomics features were extracted and selected from the region of tumor mass volume (TMV) and peritumoral tumor volume (PTV) respectively. The TMV, PTV, and combined(TM-PTV) radiomics models were established in the training cohort (*n* = 111)for the prediction of histopathological grade. Finally, a radiomics nomogram was constructed by combining the TM-PTV radiomics signature (Rad-score) and the selected clinical-MRI predictor. The ROC and calibration curves were used to determine the performance of the TMV, PTV, and TM-PTV models in the training and validation cohort (*n* = 49). The decision curve analysis (DCA) and calibration curves were used to investigate the clinical usefulness and calibration of the nomogram, respectively.

**Results:**

The TMV model, PTV model, and TM-PTV model had AUCs of 0.835, 0.879, and 0.917 in the training cohort and 0.811, 0.756, 0.896 in the validation cohort. The nomogram, including the TM-PTV signatures and peritumoral hyperintensity, achieved good calibration and discrimination with a C-index of 0.948 (95% CI, 0.906 to 0.990) in the training cohort and 0.921 (95% CI, 0.840 to 0.995) in the validation cohort. Decision curve analysis demonstrated the clinical usefulness of the nomogram.

**Conclusion:**

The proposed model based on intratumoral and peritumoral radiomics showed good performance in distinguishing low-grade from high-grade STSs.

**Supplementary Information:**

The online version contains supplementary material available at 10.1186/s40644-023-00622-2.

## Key points

• Peritumoral and intratumoral radiomics models based on FS-T2WI images had a good predictive ability for the histopathological grading of STSs.

• Peritumoral hyperintensity and radiomic scores were independently associated with histopathological grading of STSs.

• The nomogram integrating clinic features and radiomics scores demonstrated good performance for predicting the histopathological grading of STSs.

**Supplementary Information:**

The online version contains supplementary material available at 10.1186/s40644-023-00622-2.

Soft tissue sarcomas (STSs) are highly heterogeneous malignant tumors originating from mesenchymal tissue. According to the histological grading method used by the French Federation of Cancer Centers Sarcoma Group(FNCLCC) [1], STSs are divided into classes I–III based on how aggressively malignant cells manifest themselves, where grade I is a low grade and grade II, III, is a high grade. Different histopathological grades of treatment strategies and prognoses have variances [2]. Preoperative adjuvant chemoradiotherapy is recommended for high-grade STSs, which can help improve the survival rate of patients. Correspondingly, the side effects of preoperative chemoradiotherapy can be avoided if patients with low-grade STS are identified before surgery. At present, the preoperative histopathological grading diagnosis of STSs mainly relies on the core needle biopsy [3]. However, the results are easily influenced by the sampling site, the size, and the makeup of the lesion, making it impossible to do an overall assessment of the lesion [4].

The most common non-invasive technique for STS preoperative diagnosis and evaluation is magnetic resonance imaging (MRI). Although experienced radiologists can easily identify tumors from MRI, tumor heterogeneity makes grading STSs challenging [5]. Recently, there has been promising progress in the grading of STSs based on radiomic features of MRI. Zhang et al. [6] employed radiomics-based features to establish different diagnostic models to identify the grading of STSs and found the model using the support vector machine (SVM) classifier method performed best. Peeken et al. [7] established independent and combined radiomics models based on FS-T_2_WI and T1WI-enhanced sequences to predict STSs histopathological grades and found that the radiomics model based on FS-T_2_WI had the highest predictive performance for high-grade STSs. Yan et al. [6, 8] constructed a radiomics nomogram method to predict high-grade STSs, conducted model development and validation with 180 cases of STSs in two centers, and found that the nomogram based on radiomics features, T staging, and MR boundaries was superior to a single radiomics model or a clinical feature model. Although previous studies have achieved great success in differentiating pathological grades, extracting more valuable radiomics features to boost prediction accuracy remains a challenge.

However, the existing radiomics-based approaches focus on the intratumoral area and ignore the role of the peritumoral environment in STSs grading. Endothelial cells, fibroblasts, immune cells, and other cell types as well as extracellular components make up the peritumoral area, also known as the tumor microenvironment [9, 10]. The microenvironment determines many aspects of tumor behavior, including tumor progression, treatment response, and metastasis [11]. White et al. have shown that satellite-like single or clustered tumor cells that are not visible on imaging can be found beyond the tumor margins in a population of patients undergoing surgery [12]. Clinical evidence suggested the heterogeneity of STSs is not limited to tumor margins but also involves peritumoral regions [13]. As a result, the tumor’s peritumoral environment is also promising and may offer important data for the clinical evaluation of tumor invasive biological behavior. Recently, there have been some studies combining intratumoral and peritumoral radiomic features to determine the histopathological classification of clear cell renal cell carcinoma [14, 15], identify benign and malignant nodules in the lung [16, 17], predict lymph node metastasis and distant metastasis in lung cancer [18], and predict the risk of breast cancer response to chemotherapy [19], which achieved good performance.

The purpose of this study was to investigate, using a two-center dataset, the capacity of intratumoral and peritumoral radiomics signatures based on MRI to noninvasively predict STS histopathological grade.

## Materials and methods

### Patients and MRI morphologic characteristics

This two-center retrospective study’s ethical approval was provided by the two institutional review boards, and the requirement for informed consent was waived. Between June 2016 and July 2022, 160 patients with STS confirmed by pathology and met the inclusion criteria were retrospectively collected. Inclusion criteria:(1) Patients underwent surgical resection; (2)STS was diagnosed by histopathology; (3) Axial FS-T_2_WI MRI scans ≤ 2 weeks before surgery; Exclusion criteria: (1) Incomplete clinical or imaging data; (2) MRI image quality is poor, signal-to-noise ratio ≤ 1.0; (3) Development of other subsequent tumors; (4) The patient has received prior treatment, such as chemotherapy, radiation therapy, or needle biopsy.

In total, 160 STS patients were analyzed. Clinical-MRI characteristics included age, gender, location, and MRI morphological features. All images were independently reviewed by two radiologists with more than 5 years of skeletal muscle MRI experience while remaining blind to the clinical and histopathological data. Decisions on MRI findings were made through team negotiation. According to Zhao et al. [20], the following MRI morphological features were selected for comparison: (1) size (maximum diameter of tumor, < 5 cm or ≥ 5 cm); (2) margin (well- or poorly-defined); (3) signal intensity (homogeneous or heterogeneous, > 30% of the whole volume was considered heterogeneous); (4) peritumoral hyperintensity. All these MRI features were labeled as dichotomous variables and recorded using Yes or No.

**Fig. 1:**
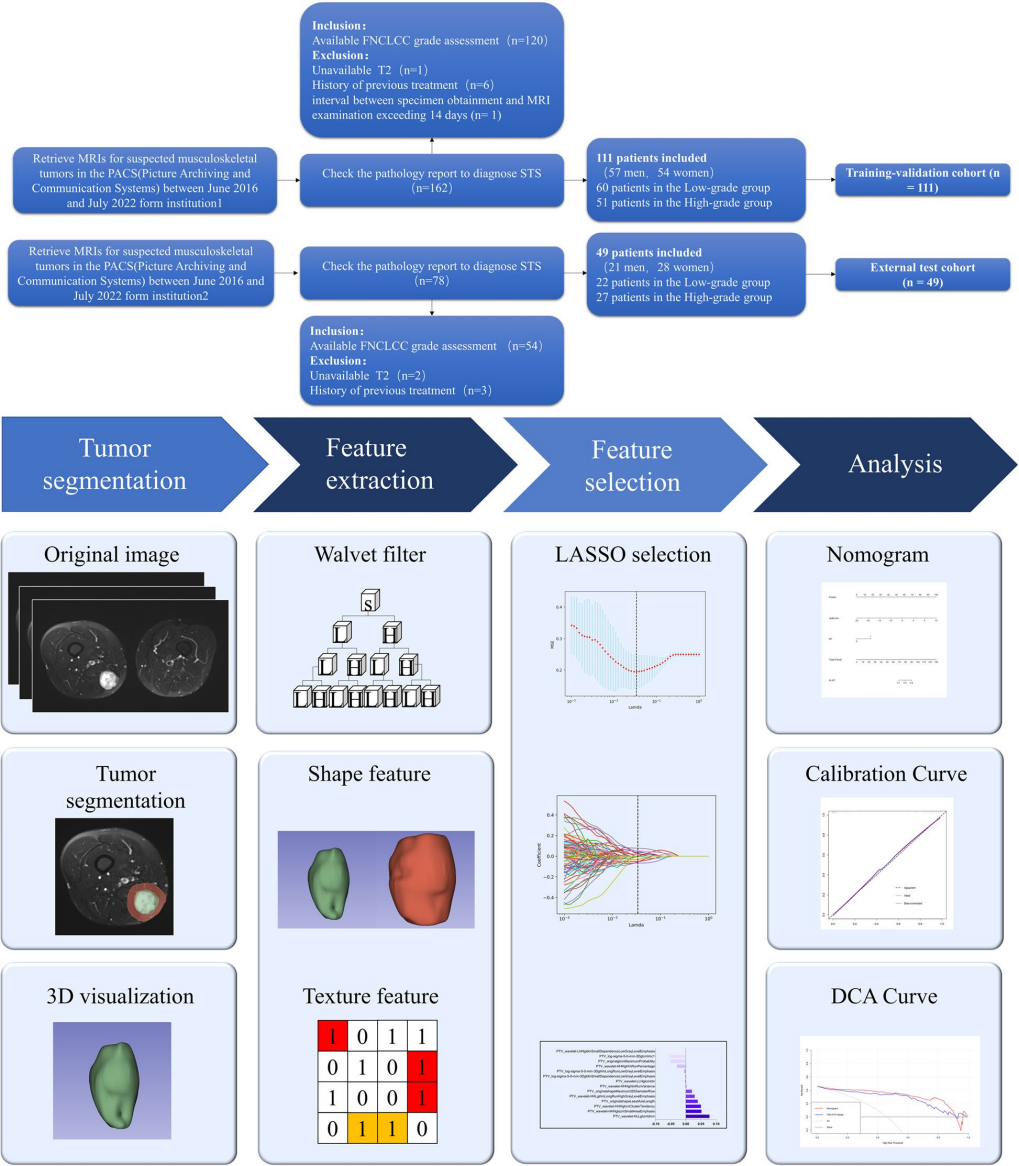
(Top) Flow chart of patient enrollment. (Bottom) Work flow of the radiomics implementation

The final histopathological results of the 160 STS patients were shown in Table 1. The FNCLCC system assigns a score to the tumor based on its mitotic index, differentiation, and amount of necrosis, and the tumor grade was calculated by adding these three scores. According to their FNCLCC tumor grade, the patients were divided into two groups: low-grade (*N* = 82) and high-grade (*N* = 78). The workflow was shown in Fig. 1.

**Table 1:** The pathologic data of the 160 STS patients

	Low-grade cohort (*n* = 82)	High-grade cohort (*n* = 78)
Histological diagnosis	Acinar rhabdomyosarcoma (*n* = 4)	Acinar rhabdomyosarcoma (*n* = 2)
	Angiosarcoma (*n* = 4)	Angiosarcoma (*n* = 3)
	Clear cell sarcoma (*n* = 6)	Clear cell sarcoma (*n* = 4)
	Epithelioid sarcoma (*n* = 5)	Epithelioid fibrosarcoma (*n* = 3)
	Fibrosarcoma (*n* = 13)	Epithelioid sarcoma (*n* = 4)
	Leiomyosarcoma (*n* = 4)	Fibrosarcoma (*n* = 13)
	Liposarcoma (*n* = 7)	Leiomyosarcoma (*n* = 4)
	Myofibroblastic sarcomas (*n* = 4)	Liposarcoma (*n* = 5)
	Myxofibrosarcoma (*n* = 9)	Myofibroblastic sarcomas (*n* = 6)
	Myxoid liposarcoma (*n* = 8)	Myxofibrosarcoma (*n* = 8)
	Pleomorphic sarcoma (*n* = 5)	Myxoid liposarcoma (*n* = 6)
	Rhabdomyosarcoma (*n* = 3)	Pleomorphic sarcoma (*n* = 3)
	Synovial sarcoma (*n* = 4)	Rhabdomyosarcoma (*n* = 2)
	Unconfirmed (*n* = 6)	Synovial sarcoma (*n* = 8)
		Undifferentiated pleomorphic sarcoma (*n* = 4)
		Unconfirmed (*n* = 3)

### MRI Acquisition

All 160 patients underwent FS-T_2_WI with Siemens Verio3.0 T, Siemens Aera 1.5T (Siemens Medical AG, Erlangen, Germany), or Philips Achia1.5t, Philips Achieva3.0T (Philips Medical Systems, Best, The Netherlands), with adapted position and coils depending on tumor size and location. The scan parameters listed below were used: TR: 2640–5000 ms; TE:30-102ms; slice gap:1 mm; slice thickness:3-4 mm; matrix:320 × 320; The field of view ranges from 200 × 200mm^2^ to 400 × 400mm^2^.

### Image segmentation and extraction

**Fig. 2:**
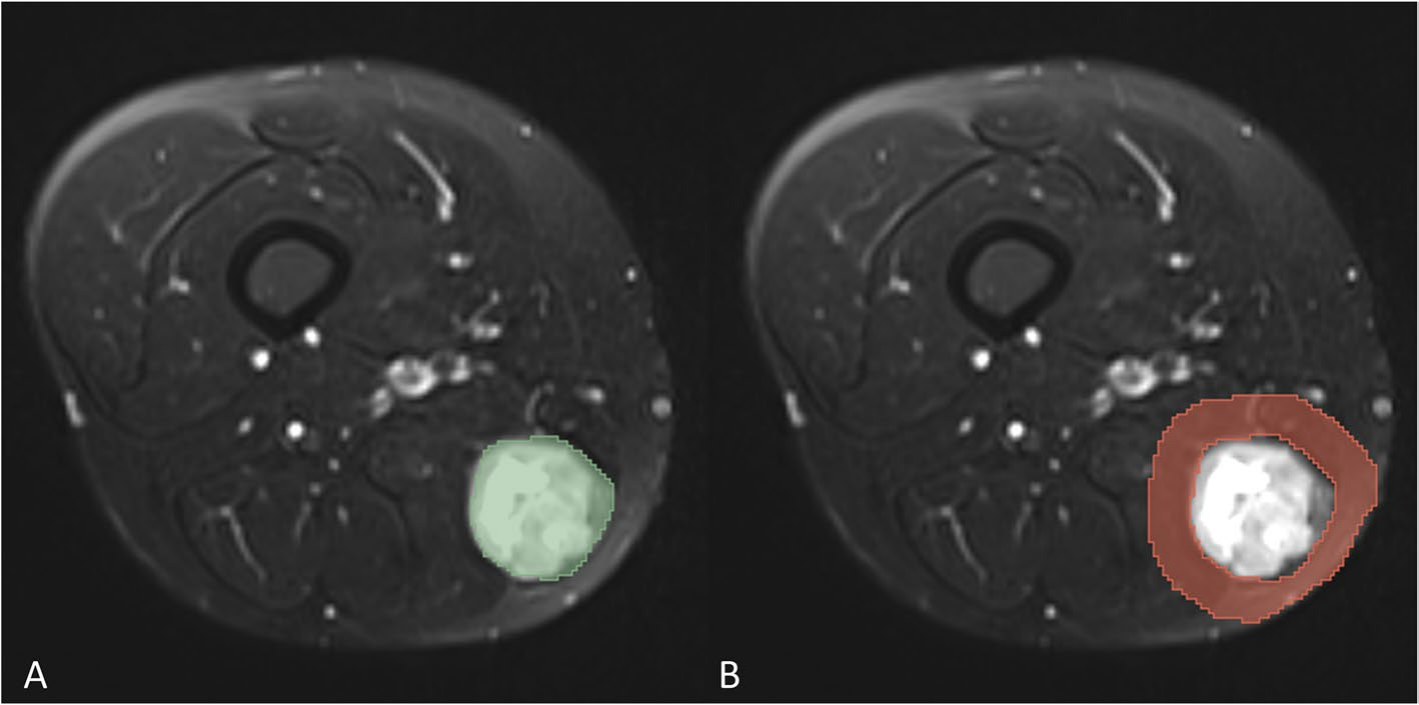
Example of delineated ROI on FS-T_2_WI mapping. A 43-years-old woman with pleomorphic sarcoma. **A** The TMV region is marked in green. **B** The PTV region is marked in red, and the air region beyond the human tissue has been removed. 1037 feature values were extracted from each of the two disjoint regions through the Slicer-radiomics extension package of 3Dslicer (Feature types, and extraction methods are included in Supplementary Material 1–2)

For image segmentation, all FS T_2_WI sequence images from patients were uploaded into 3D slicer (*version 4.10.2*, https://www.slicer.org/, *Accessed 8 June 2023*). In FS-T_2_WI images, tumor mass volume (TMV) VOIs were delineated within the margins of tumor masses, encompassing necrotic, cystic change, and hemorrhagic areas but omitting peritumoral edema. The TMV VOIs were then used as a template to construct the corresponding peritumoral tumor volume (PTV) VOIs. The PTV VOIs were generated automatically by uniformly dilating the tumor’s boundary by 10 mm in three dimensions, and adjacent air and bone were manually removed (Fig. 2). The segmentation process was independently performed by two readers (Reader1 and Reader 2) with more than five years of experience, blinded to clinical information and histopathological results. Reader 1 segmented 40 random cases to assess intra-observer reliability two weeks later. Additionally, Reader 2 completed the same 40 random cases to assess inter-observer reliability. Intra- and inter-class Dice coefficients were calculated to assess the stability of delineated VOIs. Features extracted from VOIs with ICCs greater than 0.75 were retained for subsequent investigation.

Preprocessing procedures were used to reduce the bias of the features and to counteract the intensity inhomogeneity caused by different imaging protocols before radiomics feature extraction. All VOIs were normalized and resampled to the same resolution (1 mm×1 mm×1 mm) to eliminate data heterogeneity. The limitation of dynamics to µ ± 3σ (µ gray level mean, σ standard deviation) was used to minimize the influence of contrast and brightness variation [21].

Radiomics features were extracted via the Slicer-Radiomics extension in 3D Slicer which enables processing and extraction of radiomic features from medical image data using a large panel of engineered hard-coded feature algorithms by accessing PyRadiomics (https://github.com/AIM-Harvard/pyradiomics, *Accessed 8 June 2023*) [22]. The detailed operation of extracting features is shown in the Supplementary material (M1-2) 0.1037 radiomics features were extracted from each VOI of TMV and PTV, including first-order statistics(first-order), shape-based(3D) features, shape-based (2D) features, grey-level cooccurrence matrix (GLCM), grey-level run length matrix (GLRLM), grey-level size zone matrix (GLSZM), neighboring grey tone difference matrix (NGTDM), grey-level dependence matrix (GLDM), and wavelet decomposition features. Before further analysis, all the extracted radiomics features of TMV, and PTV were normalized by Z score transformation [23] and ComBat compensation [24] to eliminate the differences in the value scales of the data and remove the batch effects derived from multiple sources of variability caused by different scanners and protocols.

### Feature selection

Feature selection was conducted using python software (*version 3.8.8*, https://www.python.org/, *Accessed 8 June 2023*), which is mainly implemented by calling the scikit-learn library, a widely used Python library for machine learning and data science [25]. In this step “levene,” “ttest” and “LassoCV” function will be used to select features. A two-step feature selection methodology was performed for the training - validation cohort. Firstly, A *t*-test was used to filter out the features that were significantly different between the low-grade and high-grade groups. Secondly, the least absolute shrinkage and selection operator (LASSO) method was applied to select the most powerful features in the training - validation cohort and selected non-zero coefficients based on 10 cross-validation. All codes and additional details can be found online (https://github.com/mystic1602/radiomics, *Accessed 8 June 2023*).

### Model construction, rad-score building

To assess the feasibility and promise of the FS-T_2_WI-based peritumoral radiomics signature for detecting low- and high-grade STSs, the following 3 types of radiomic signatures were extracted: (1) radiomics signatures from TMV features; (2) radiomics signatures from PTV features; (3) radiomics signatures from the merged features of TMV and PTV (TM-PTV).TMV and PTV radiomics signatures were created utilizing the same approach described in the “Radiomics features extraction” and “Feature selection” subsections. TM-PTV features were created by combining TVM and PTV features, and the statistically significant features were chosen using the approach described in the “Feature selection” part.

Prediction models of 3 types of radiomics signatures were created using logistic regression, and three types of radiomics signatures were fed into the the GridSearchCV to establish an ideal parameter configuration [26]. In the external test cohort, their predictive performance was assessed utilizing the area under the curve (AUC) of receiver operating characteristic (ROC) curve analysis. The AUC of each model was evaluated first, and the best model was picked for further investigation. The Rad-score was then calculated using a LASSO logistic regression model based on the best type of radiomics signature.

### Development and validation of Nomogram

Univariate and multivariate logistic regression analyses were used to select clinical features and the Rad-score, and a nomogram was constructed based on the independent risk factors in the multivariate study. The model’s discriminative capacity was evaluated using Harrell’s concordance (C-index) with confidence intervals of 95% for both cohorts. The calibration curve was plotted to investigate the model’s predictive accuracy. To assess clinical usefulness, decision curve analysis (DCA) was used to calculate the net benefit of the nomogram model in training and validation groups.

### Statistical analysis

Statistical analyses were performed by GraphPad Prism (*version 9.4*, https://www.graphpad.com/, *Accessed 8 June 2023*), and R software (*version 3.6.2*, http://www.Rproject.org, *Accessed 8 June2023*). When comparing clinical data, The *t*-test or *Mann-Whitney U* test was used for continuous variables and *Fisher’s exact* test for categorical variables. The plot nomograms and calibration curves using the “RMS” software package and the DCA curve were drawn using the “RMDA” software package. Two-sided *P* < 0.05 was considered statistically significant for all tests.

## Results

### Clinical data: patient and MRI morphological characteristics

One hundred sixty patients were recruited for this study. In the training cohort, there were 51 high-grade (grade II-III) patients and 60 low-grade (grade I) patients. In the validation cohort, there were 27 high-grade (grade II-III) patients and 22 low-grade (grade I) patients. The detailed clinical and MRI morphologic characteristics of the study population were listed in Table 2. There was no significant difference in age, sex, tumor location, peritumoral hyperintensity, size, and margin between the two cohorts. There were significant differences in signal uniformity between the two cohorts (*p* < 0.05).

**Table 2:** Demographic data of patients in the training and validation cohorts

Variable	Training cohort (*n* = 111)			Validation cohort (*n* = 49)			*P*
	Low-grade group	High-grade group	*P*	Low-grade group	High-grade group	*p*	
**Age (Years, Mean ± SD)**	51.57 ± 14.09	47.65 ± 14.90	0.178^a^	56.27 ± 20.46	48.15 ± 14.68	0.165^a^	0.660^a^
**Sex, n (%)**			0.7053^b^^*^			0.246^b^	0.392^b^
Male	32(0.53)	25(0.49)		7(0.32)	14(0.52)		
Female	28(0.47)	26(0.51)		15(0.68)	13(0.48)		
**Tumor Location**			0.570^b^			0.567^b^	0.864^b^
Limb	28(0.47)	27(0.53)		9(0.41)	14(0.52)		
Body	32(0.53)	24(0.47)		13(0.59)	13(0.48)		
**Peritumoral Hyperintensity**			< 0.0001^b*^			0.001^b*^	> 0.999^b^
No	38(0.63)	9(0.18)		15(0.68)	5(0.19)		
yes	22(0.37)	42(0.82)		7(0.32)	22(0.81)		
**Size**			0.306^b^^*^			0.388^b^	0.111^b^
< 5 cm	44(0.73)	32(0.82)		14(0.64)	13(0.48)		
≥ 5 cm	16(0.27)	19(0.18)		8(0.36)	14(0.52)		
**Margin**			0.329^b^			0.3931^b^	0.224^b^
Well-defined	20(0.33)	22(0.43)		9(0.41)	15(0.56)		
Poorly-defined	40(0.67)	29(0.57)		13(0.59)	12(0.44)		
**Signal Intensity**			> 0.999^b^			0.260^b^	0.0009^b^
Homogeneous	42(0.70)	35(0.69)		11(0.50)	9(0.33)		
Heterogeneous	18(0.30)	16(0.31)		11(0.50)	18(0.67)		

^*^P-value < 0.05

^a^Mann-Whitney U test

^b^Fisher’s exact test

### Feature selection

The intra-class Dice coefficient of TMV was 0.908 ± 0.032, and the inter- was 0.876 ± 0.057; the intra-class Dice coefficient of PTV was 0.842 ± 0.060, and the inter- was 0.816 ± 0.050 (Supplementary material F1). Features for TMV and PTV were reduced to 845 and 688 respectively after excluding features with ICC less than 0.75. The reserved features were analyzed using a *t*-test (*p* < 0.05) to identify features with significant between-group differences between low- and high-grade STSs. Following statistical analysis-based feature selection, three radiomics feature subsets were obtained: (1) 72 significant TMV features, (2) 322 significant PTV features, and (3) 394 significant TMV and PTV features (TM-PTV). Finally, LASSO was used to pick all of the significant features in each feature subset, 8, 14, and 18 discriminative features were chosen, respectively, to create a peritumoral radiomics signature for malignancy grading from TMV, PTV, and TM-PTV. The details of these features were shown in Supplementary material M3-4 and F2.

### Performance of radiomics signatures

**Fig. 3:**
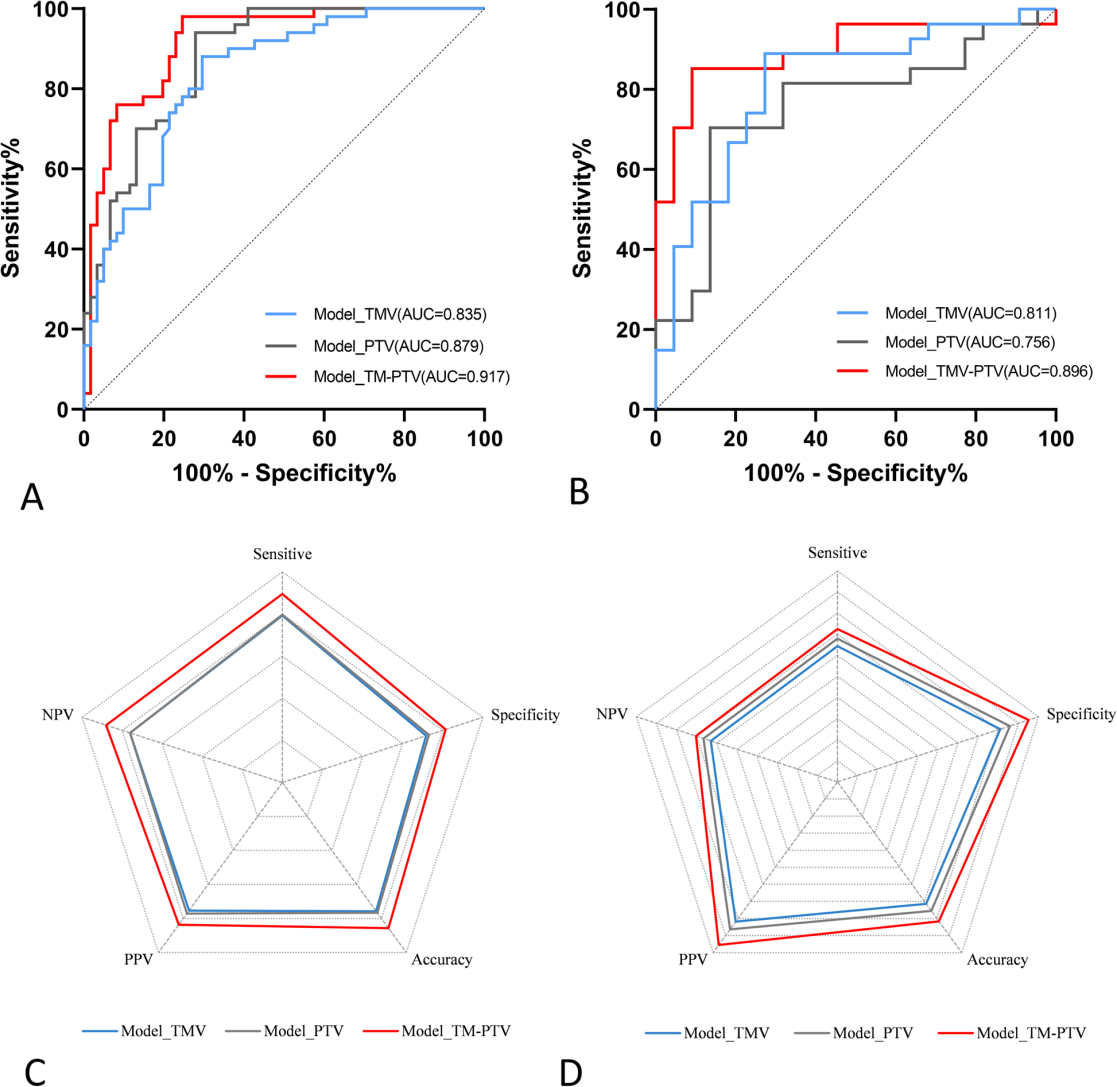
The ROCs and radar chart performance of Model_TMV, Model_PTV, and Model_TM-PTV in training (**A**, **C**) and validation (**B**, **D**) cohorts, respectively. AUC: area under the ROC curve. NPV: negative predictive value; PPV: positive predictive value

The AUC of the TM-PTV model was higher than that of the TMV model or PTV model in both the training and validation cohorts. All of the models in the training group had similar performance. However, in the validation cohort, the sensitive, specificity, accuracy, PPV and NPV of the TMV-PTV model is higher than other models (Fig. 3; Table 3).

**Table 3:** Performance of LASSO models in train and validation cohort

Model	Sensitive	Specificity	Accuracy	PPV	NPV
**Train cohort**					
Model_TMV	0.79	0.72	0.76	0.75	0.76
Model_PTV	0.80	0.73	0.77	0.77	0.76
Model_TM-PTV	0.89	0.81	0.86	0.84	0.88
**Validation cohort**					
Model_TMV	0.64	0.81	0.71	0.82	0.63
Model_PTV	0.68	0.86	0.76	0.86	0.67
Model_TM-PTV	0.72	0.95	0.82	0.95	0.70

*PPV* positive predictive value, *NPV* negative predictive value

### Rad-score building, identification of Independent risk factors

The TM-PTV model displayed better performance than the other two models. As a result, a LASSO logistic regression model was used to calculate the radiomics score (Rad-score) based on TM-PTV. Supplementary material M4 and F3 illustrate the details of the Rad-score computation formula. A logistic regression analysis with backward stepwise selection identified the Rad-score and peritumoral hyperintensity as independent predictors (Table 4), which were then used to create a personalized prediction nomogram.

**Table 4:** Result of Univariate Logistic Regression and Multivariable Logistic Regression

Variable	Univariate Logistic Analysis			Multivariable Logistic Analysis			
	OR	95%CI	*P*	Coefficient	OR	95%CI	*P*
**Age**	0.980	[0.954;1.006]	0.139				
**Sex**	1.189	[0.563;2.520]	0.650				
**Tumor Location**	0.778	[0.366;1.642]	0.510				
**Peritumoral Hyperintensity**	8.061	[3.424;20.61]	< 0.0001^*^	2.856	17.38	[4.751;81.16]	< 0.0001
**Size**	1.633	[0.731;2.692]	0.2331				
**Margin**	0.611	[0.2787;1.325]	0.214				
**Signal Intensity**	1.067	[0.472;2.400]	0.876				
**Rad-Score**	2.279	[1.729;3.266]	< 0.0001^*^	0.876	2.401	[1.781;3.550]	< 0.0001^*^

*OR* odd ratio, *CI *confidence interval

^*^P-value < 0.05

### Development and validation of nomogram

**Fig. 4:**
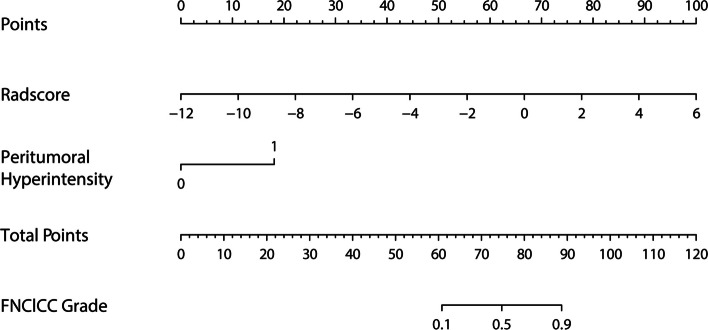
Nomogram used to distinguish between low- and high-grade levels in soft tissue sarcomas. Development of the radiomics-clinical nomogram, which includes the Rad-score within the second row, and “Peritumoral hyperintensity” within the third row are summed to give the “total points,” which are marked on the “Total points” row. The probability of high grad is read off from the scale in the last row by vertically drawing a line from the total points

**Fig. 5:**
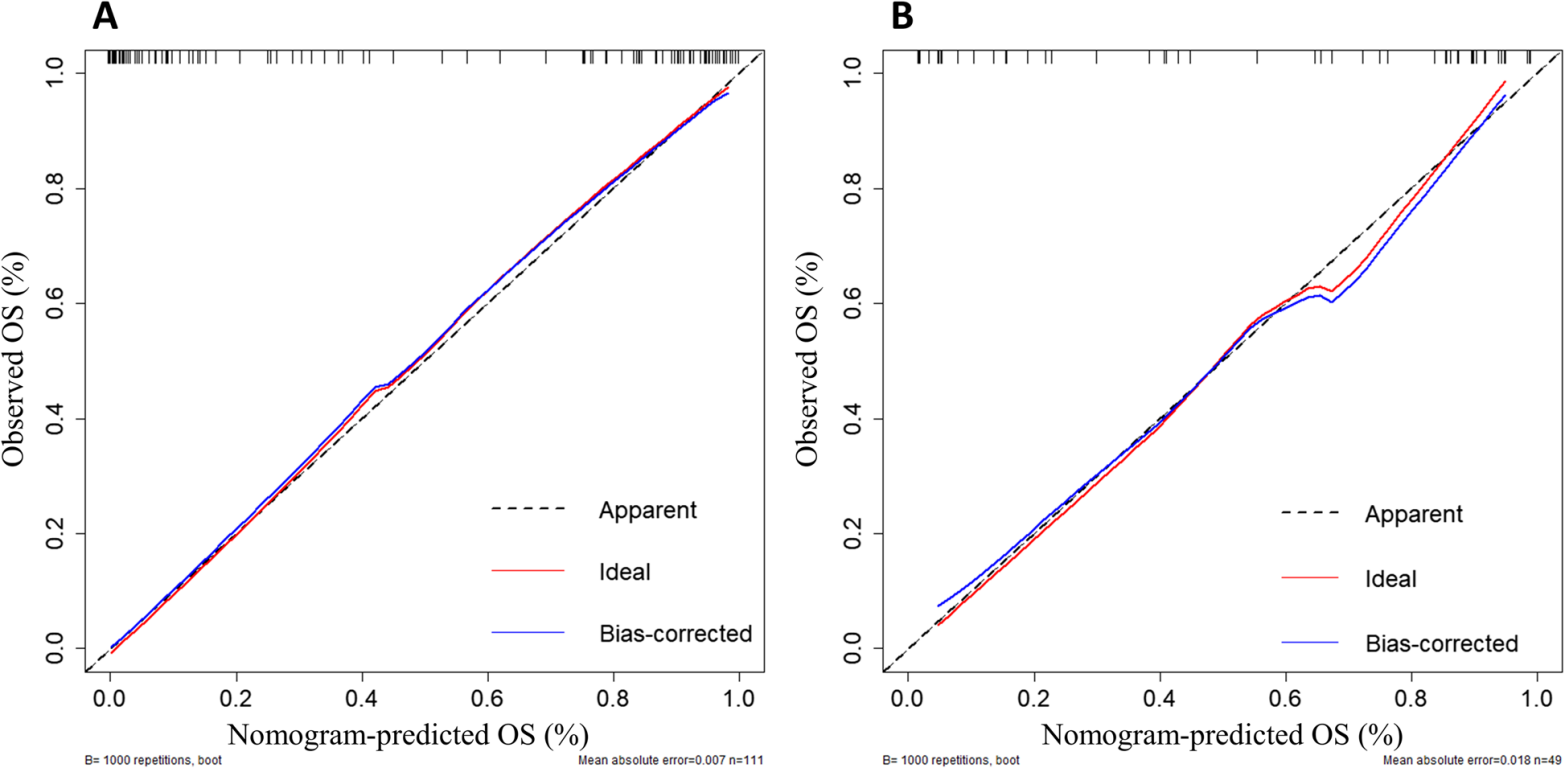
Calibration curve of the nomogram in the training cohort (**A**) and validation cohort (**B**). The blue line represents the perfect prediction of the ideal model, and the red line represents the performance of the model. The Grey line represents corrected predictive performance. The closer the red line and the blue line are, it means that the predicted results are in good agreement with the actual results, and the prediction ability is better

A model containing the above independent predictors was shown in a nomogram (Fig. 4). The model showed a favorable C-index of 0.948 (95% CI, 0.906 to 0.990) in the training cohort and0.921 (95% CI, 0.840 to 0.995) in the validation cohort. Calibration curves of radiomics nomograms used to predict the histopathological grade of STSs showed good agreement between predicted and observed outcomes for both cohorts (Fig. 5). The Hosmer-Lemeshow test showed no statistically significant difference between the calibration curve and the ideal curve (training cohort: χ^2^ = 8.275, *p* = 0.407, validation cohort: χ^2^ = 9.790, *p* = 0.280), indicating no deviation from a perfect fit.

**Fig. 6:**
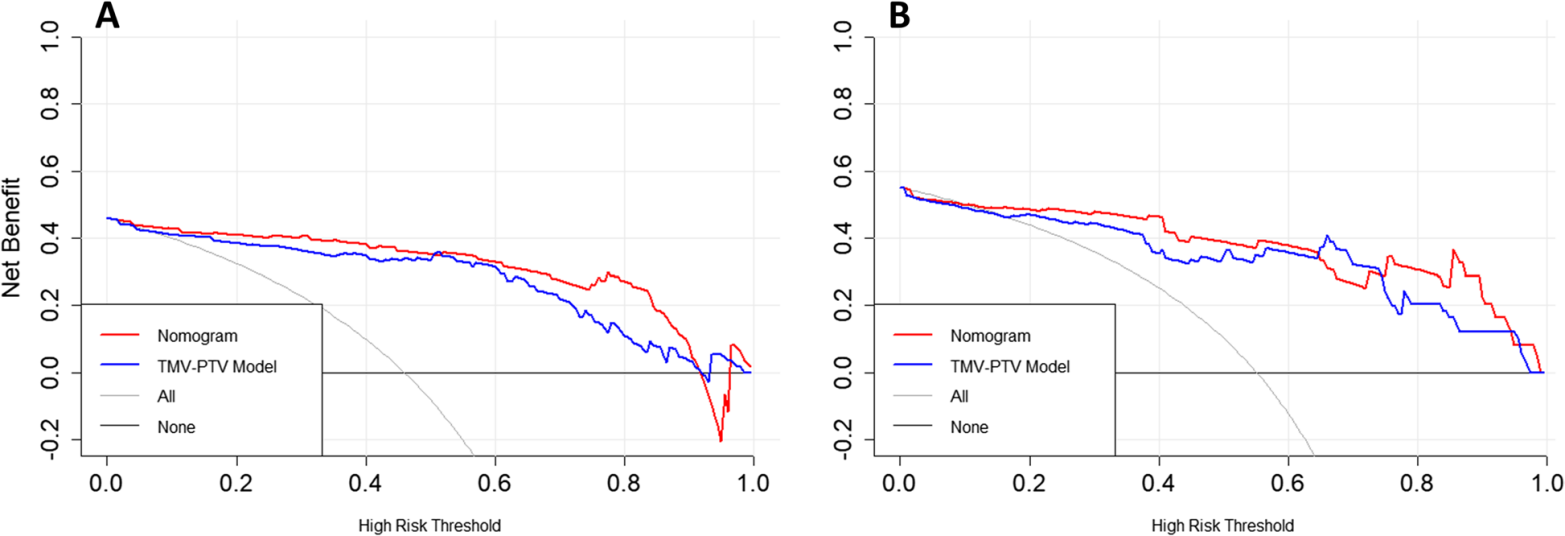
Decision curve analysis (DCA). The y axis represents the net benefit, which was determined by calculating the difference between the expected benefit and the expected harm associated with each proposed model [net benefit = true-positive rate (TPR) – (false-positive rate (FPR)× weighting factor), where the weighting factor = threshold probability/ (1-threshold probability)]. The gray line represents the assumption that all tumors were histopathological high-grade (the treat-all scheme). The black line represents the assumption that all tumors were histopathological low-grade expression (the treat-none scheme). **A **DCA in the training cohort. For threshold probabilities from 4 to 90%, using the nomogram to predict the histopathological grade added more benefit than using the radiomics model. **B** DCA in the validation cohort. for threshold probabilities ranging from 8–64% and greater than 75%, using the nomogram to predict the histopathological grade added more benefit than using the TM-PTV model

The DCA of the TM-PTV model (based on Rad-score) and nomogram model is shown in Fig. 6. In the training cohort, using the nomogram to predict histopathological grade added more benefits than using the TM-PTV model for threshold probabilities from 4 to 90%. In the validation cohort, using the nomogram to predict histopathological grade added more benefit than using the TM-PTV model for threshold probabilities ranging from 8 to 64% and greater than 75%.

The DCA revealed that the net benefit of the nomogram was higher than that of the TM-PTV model, suggesting that the predicting strategy based on nomogram has better clinical utility.

## Discussion

A radiomics nomogram was developed based on the combination of intra-peritumoral radiomic features and peritumoral hyperintensity, which successfully differentiated low-grade and high-grade STSs, and its recognition performance was higher than the TM-PTV model, and showed good calibration in both groups, suggesting that it may be a promising tool for clinical strategy. To the best of our knowledge, this is the first study to develop a model based on intratumoral and peritumoral radiomics to predict the histopathological grade of STSs. The present study found peritumoral hyperintensity and Rad-score were independent risk factors for predicting histopathological grade. Peritumoral hyperintensity on FS-T_2_WI may be caused by surrounding tissue edema, inflammatory cell infiltration, angiogenesis, and other changes [27]. It has been widely recognized as a significantly poor prognostic factor for STS [28].

Previous studies have reported on an MRI-based radiomics model for predicting STS histopathological grade [6, 8]. Zhang et al. [6] developed an MRI-based radiological model with an AUC of 0.92 in predicting histopathological grade. Yan et al. [8] developed an MRI-based radiomics nomogram for predicting the grade and demonstrated good calibration and good clinical utility. These two available studies were all based on TMV models. However, Significant clinical evidence suggests the heterogeneity of STSs is not limited to tumor margins but also involves peritumoral regions [13]. White et al. have demonstrated in a population of patients undergoing surgery, satellite-like single or clustered tumor cells that are not visible on imaging can be found beyond the tumor margins [12].In our study, the TMV, PTV, and TM-PTV models demonstrated outstanding discrimination in both the training and validation cohorts. Compared with the TMV model, the combination of the TMV and PTV(PT-TMV) model significantly improved the AUC, accuracy, sensitivity, specificity, PPV, and NPV of the predictive model. This result indicated that the combination of peritumoral features provides more information about the tumor microenvironment, which can reflect the biological behavior of the tumor better.

Our study used a 10 mm extension from the lesion, which is based on the current standard for STSs surgical margins. To protect critical neurovascular structures or bones, a margin aiming at 10 mm is the minimum appropriate width to be considered acceptable according to National Comprehensive Cancer Network (NCCN) guidelines [29]. Previous peritumoral radiomics studies of lung cancer [30] and glioma [14] have shown that the closer the peritumoral region is to the intratumoral region, the more information it contains. As the expansion distance increases, more normal soft tissue is incorporated into the region of interest (ROI), resulting in a smaller difference in peritumoral tissue heterogeneity. Radiomics features extracted from a 10 mm peritumoral ROI were most likely to provide important information for predicting histopathological grade for STS. In this study, the radiomics model was constructed based on a single FS-T2WI sequence, which was currently the most widely used sequence for STS radiomics. Peeken’s study found the FS-T2WI-based model showed better reproducibility compared with the T1WI model and the combined T1 and T2WI models [7]. Importantly, the present study focused on the efficacy of radiomics models of the tumor itself and the tumor microenvironment. However, most lesions have unclear borders on T1WI, and image segmentation is difficult, which may affect the division of intratumoral and peritumoral ROIs. In addition, extracting a large number of features from many sequences tends to increase the risk of model overfitting [31].

Radiomics features are sensitive to all the acquisition conditions including MR protocols, scanners, and MR adjustments. To lower bias and variance, We performed several preprocessing methods, In particular, the ComBat compensation,which is easy to apply and is suitable for retrospective information analysis [24], eliminates batch effects due to multi-source variation caused by different scanners and protocols in multi-center radiation analysis while preserving the excellent properties of its texture patterns and has been used in previous reports to improve reproducibility between different centers [32]. Therefore, the key to addressing the heterogeneity of acquisition conditions may lie in adequate preprocessing and consistent scanning parameters.

When dealing with radiomics, the segmentation method has a large impact on the reproducibility and reliability of the radiomics signature [33]. The current image segmentation methods include manual, semiautomatic, and fully automatic. Manual segmentation is prone to interobserver variability, which may hinder the reproducibility of radiomics analyses. Automatic segmentation techniques based on deep learning are the mainstream of the current research field and are currently being considered for clinical trials, showing improvements in image classification prediction and recognition tasks [34]. While results from automatic image segmentation are promising, errors due to contrast blur and biased fields are common and often require manual correction to ensure accuracy [35]. We tried to use U-net neural network for fully automatic segmentation of lesions, but because STS comes from various types of tissues, shapes, and positions are not fixed, the automatic segmentation of lesions was not effective. Therefore, this study still used the traditional manual segmentation method. Although both the intra-observer and inter-observer ICC coefficients were high, this was a hugely time-consuming process, which was a limitation of this study. In the future, in-depth research was expected to break through the limitations of image segmentation. This study has several limitations that need to be addressed. First, our study was retrospective, so despite our strict criteria, there is a potential selection bias. Second, our data came from two institutions using similar but different scanners and protocols. Therefore, the resampling methodology and the combat compensation method were adopted to reduce the difference in image specifications to improve the stability of features and different models. Third, multimodal MR radiomics may have better potential, such as DWI. Finally,this study did not include all histopathological types of STS because our study was retrospective and limited by the number of patients. Therefore, the sample needs to be enlarged in future research to improve the generalization ability of the model.

In conclusion, peritumoral radiomics features can provide complementary information to intratumoral regions to predict the histopathological grade of STS. Such quantitative radiomics prognostic models of STS may potentially be useful for precision medicine.

### Supplementary Information

**Additional file 1: Material 1.** Detailed information about extracted radiomics features. **Material 2.** Image Types were used to extract features from. **Material 3.** Features screened by the LASSO method. **Material 4.** Calculation formula of Rad-score. **Figure 1.** The Dice coefficient of TMV and PTV. **Figure 2.** Detailed information about feature extract by python. **Figure 3.** Rad-score for each patient in each cohort.

## Data Availability

The datasets used and/or analyzed during the current study are available from the corresponding author upon reasonable request. All codes and additional details can be found online (https://github.com/mystic1602/radiomics, Accessed 8 June 2023).

## Abbreviations

AUC: Area Under Curve
DCA: Decision Curve Analysis
DWI: Diffusion Weighted Imaging
FNCLCC: French Federation of Cancer Centers Sarcoma Group
FST2WI: Fat-Saturated T2-Weighted
ICC: Intraclass Correlation Coefficient
LASSO: Least Absolute Shrinkage and Selection Operator
MRI: Magnetic Resonance Imaging
PTM: Peritumoral Tumor Volume
ROC: Receiver Operating Characteristic Curve
ROI: Region of Interest
STSs: Soft Tissue Sarcomas
SVM: Support Vector Machine
TM-PTV: Combined TMV and PTV
TMV: Tumor Mass Volume
VOI: Volume of Interest
