# Supplementary material for: Intratumoral and peritumoral MRI-based radiomics prediction of histopathological grade in soft tissue sarcomas: a two-center study
Source: Cancer Imaging. 2023 Oct 26;23:103. doi: 10.1186/s40644-023-00622-2 (PMC10601231; doi:10.1186/s40644-023-00622-2)
Supplement: Supplementary file 1 — Additional file 1: Material 1. Detailed information about extracted radiomics features. Material 2. Image Types were used to extract features from. Material 3. Features screened by the LASSO method. Material 4. Calculation formula of Rad-score. Figure 1. The Dice coefficient of TMV and PTV. Figure 2. Detailed information about feature extract by python. Figure 3. Rad-score for each patient in each cohort. [file 40644_2023_622_MOESM1_ESM.docx]

**Supplementary Material**

**Material 1 Detailed information about extracted radiomics features**

**Material 2 Image Types were used to extract features from**

**Material 3 Features screened by the LASSO method**

**Material 4 Calculation formula of Rad-score**

**Figure 1 The Dice coefficient of TMV and PTV**

**Figure 2 Detailed information about feature extract by python**

**Figure 3 Rad-score for each patient in each cohort**

**Detailed information about extracted radiomics features**

1. **19 First-order (FO) features** Energy, TotalEnergy, Entropy, Minimum, 10Percentile, 90Percentile, Maximum, Mean, Median, InterquartileRange, Range, MeanAbsoluteDeviation, RobustMeanAbsoluteDeviation, RootMeanSquared, StandardDeviation, Skewness, Kurtosis, Variance, Uniformity.
2. **16 Shape features (3D)**: MeshVolume, VoxelVolume, SurfaceArea, SurfaceVolumeRatio, Sphericity, Compactness1, Compactness2, SphericalDisproportion, Maximum3DDiameter, Maximum2DDiameterSlice, Maximum2DDiameterColumn, Maximum2DDiameterRow, MajorAxisLength, MinorAxisLength, LeastAxisLength, Elongation, Flatness.
3. **10 Shape features (2D):**MeshSurface, PixelSurface, Perimeter, PerimeterSurfaceRatio, Sphericity, SphericalDisproportion, MaximumDiameter, MajorAxisLength, MinorAxisLength, Elongation.
4. **24 Gray Level Co-occurrence Matrix (GLCM) Features:** Autocorrelation, JointAverage, ClusterProminence, ClusterShade, ClusterTendency, Contrast, Correlation, DifferenceAverage, DifferenceEntropy, DifferenceVariance, Dissimilarity, JointEnergy, Imc1, Imc2, Idm, MCC, Idmn, Id, Idn, InverseVariance, MaximumProbability, SumAverage, SumEntropy, SumSquares.
5. **16 Gray Level Size Zone Matrix (GLSZM) Features:** SmallAreaEmphasis, LargeAreaEmphasis, GrayLevelNonUniformity, GrayLevelNonUniformityNormalized, SizeZoneNonUniformity, SizeZoneNonUniformityNormalized, ZonePercentage, GrayLevelVariance, ZoneVariance, ZoneEntropy, LowGrayLevelZoneEmphasis, HighGrayLevelZoneEmphasis, SmallAreaLowGrayLevelEmphasis, SmallAreaHighGrayLevelEmphasis, LargeAreaLowGrayLevelEmphasis, LargeAreaHighGrayLevelEmphasis.
6. **16 Gray Level Run Length Matrix (GLRLM) Features:** hortRunEmphasis, LongRunEmphasis, GrayLevelNonUniformity, GrayLevelNonUniformityNormalized, RunLengthNonUniformity, RunLengthNonUniformityNormalized, RunPercentage, GrayLevelVariance, RunVariance, RunEntropy, LowGrayLevelRunEmphasis, HighGrayLevelRunEmphasis, ShortRunLowGrayLevelEmphasis, ShortRunHighGrayLevelEmphasis, LongRunLowGrayLevelEmphasis, LongRunHighGrayLevelEmphasis.
7. **5 Neighbouring Gray Tone Difference Matrix (NGTDM) Features:** Coarseness, Contrast, Busyness, Complexity, Strength.
8. **14 Gray Level Dependence Matrix (GLDM)** Features: SmallDependenceEmphasis, LargeDependenceEmphasis, GrayLevelNonUniformity, DependenceNonUniformity, DependenceNonUniformityNormalized, GrayLevelVariance, DependenceVariance, DependenceEntropy, LowGrayLevelEmphasis, HighGrayLevelEmphasis, SmallDependenceLowGrayLevelEmphasis, SmallDependenceHighGrayLevelEmphasis, LargeDependenceLowGrayLevelEmphasis, LargeDependenceHighGrayLevelEmphasis.

**Image Types were used to extract features from**

These are the image types (either the original image or derived images using filters) that can be used to extract features from.

1. **Original:** No filter applied.
2. **Wavelet:** Wavelet filtering, yields 8 decompositions per level (all possible combinations of applying either a High or a Low pass filter in each of the three dimensions).
3. **LoG:** Laplacian of Gaussian filter, edge enhancement filter. Emphasizes areas of gray level change, where sigma defines how coarse the emphasised texture should be. A low sigma emphasis on fine textures (change over a short distance), where a high sigma value emphasises coarse textures (gray level change over a large distance). In this study the LoG kernel sizes were set to 4mm and 5mm.

**Features screened by LASSO method**

| **Model** | **Radiomics Feature** | **Radiomic Group** | **Feature Class** | **coefficients** |
| --- | --- | --- | --- | --- |
| **TMV** |  |  |  |  |
|  | Diametercolumn | Maximum2D | Originalshape | 0.02903 |
|  | Smallarealowgraylevelemphasis | Glszm | Log-Sigma-5-0-Mm-3D | -0.05699 |
|  | Zoneentropy | Glszm | Wavelet-LHL | 0.059924 |
|  | Energy | Firstorder | Wavelet-LHH | 0.07374 |
|  | Smallareaemphasis | Glszm | Wavelet-LHH | 0.042542 |
|  | Imc1 | Glcm | Wavelet-HLL | 0.034129 |
|  | Kurtosis | Firstorder | Wavelet-HHL | -0.11334 |
|  | Smallareaemphasis | Glszm | Wavelet-HHH | 0.041594 |
| **PTV** |  |  |  |  |
|  | Leastaxislength | Shape | Original | 0.039379 |
|  | Maximum2DDiameterRow | Shape | Original | 0.020071 |
|  | Maximumprobability | Glcm | Original | -0.051326 |
|  | Imc1 | Glcm | Log-Sigma-5-0-Mm-3D | -0.055874 |
|  | Smalldependencelowgraylevelemphasis | Gldm | Log-Sigma-5-0-Mm-3D | -0.001495 |
|  | Longrunlowgraylevelemphasis | Glrlm | Log-Sigma-5-0-Mm-3D | -0.006036 |
|  | Idn | Glcm | Wavelet-LLH | 0.000298 |
|  | Smalldependencelowgraylevelemphasis | Gldm | Wavelet-LHH | -0.066077 |
|  | Idmn | Glcm | Wavelet-HLL | 0.077968 |
|  | Longrunhighgraylevelemphasis | Glrlm | Wavelet-HHL | 0.029182 |
|  | Clustertendency | Glcm | Wavelet-HHH | 0.049946 |
|  | Runpercentage | Glrlm | Wavelet-HHH | -0.029443 |
|  | Runvariance | Glrlm | Wavelet-HHH | 0.00183 |
|  | Smallareaemphasis | Glszm | Wavelet-HHH | 0.051617 |
| **TMV-PTV** |  |  |  |  |
| TMV | Smallarealowgraylevelemphasis | Glszm | Log-Sigma-5-0-Mm-3D | -0.01815 |
| TMV | Zoneentropy | Glszm | Wavelet-LHL | 0.010174 |
| TMV | Energy | Firstorder | Wavelet-LHH | 0.005353 |
| TMV | Kurtosis | Firstorder | Wavelet-HHL | -0.11149 |
| TMV | Smallareaemphasis | Glszm | Wavelet-HHH | 0.037906 |
| PTV | Leastaxislength | Shape | Original | 0.034597 |
| PTV | Maximum2DDiameterRow | Shape | Original | 0.017484 |
| PTV | Maximumprobability | Glcm | Original | -0.01692 |
| PTV | Zoneentropy | Glszm | Original | 0.003285 |
| PTV | Imc1 | Glcm | Log-Sigma-5-0-Mm-3D | -0.05329 |
| PTV | Idn | Glcm | Wavelet-LLH | 0.003958 |
| PTV | Smalldependencelowgraylevelemphasis | Gldm | Wavelet-LHH | -0.05596 |
| PTV | Idmn | Glcm | Wavelet-HLL | 0.088464 |
| PTV | Longrunhighgraylevelemphasis | Glrlm | Wavelet-HHL | 0.029062 |
| PTV | Clustertendency | Glcm | Wavelet-HHH | 0.035528 |
| PTV | Runpercentage | Glrlm | Wavelet-HHH | -0.02487 |
| PTV | Smallareaemphasis | Glszm | Wavelet-HHH | 0.046863 |
| PTV | Imc2 | Glcm | Wavelet-LLL | 0.017538 |

**Calculation formula of Rad-score**

**Rad-score =** -0.3981086678113797 * TMV-log-sigma-5-0-mm-3DglszmSmallAreaLowGrayLevelEmphasis + 0.4299640489904359 * TMV-wavelet-LHLglszmZoneEntropy + 0.1239700692706366 * TMV-wavelet-LHHfirstorderEnergy + -1.7496790071722195 * TMV-wavelet-HHLfirstorderKurtosis + 0.2305944693495463 * TMV-wavelet-HHHglszmSmallAreaEmphasis + -0.06531268097973612 * PTV-originalshapeLeastAxisLength + 0.21033226926548865 * PTV-originalshapeMaximum2DDiameterRow + -0.2589711124611504 * PTV-originalglcmMaximumProbability + -0.9544730127379996 * PTV-log-sigma-5-0-mm-3DglcmImc1 + 0.13959828816409353 * PTV-wavelet-LLHglcmIdn + -0.3573650483161069 * PTV-wavelet-LHHgldmSmallDependenceLowGrayLevelEmphasis + 0.014983549962018238 * PTV-wavelet-LHHglrlmShortRunEmphasis + 1.2903510349812775 * PTV-wavelet-HLLglcmIdmn + 0.41161081407071787 * PTV-wavelet-HHLglrlmLongRunHighGrayLevelEmphasis + 0.3152751921986684 * PTV-wavelet-HHHglcmClusterTendency + -0.23434960227351936 * PTV-wavelet-HHHglrlmRunPercentage + 0.5338033423100623 * PTV-wavelet-HHHglszmSmallAreaEmphasis + 0.23623291423952344 * PTV-wavelet-LLLglcmImc2

**Figure 1**

**
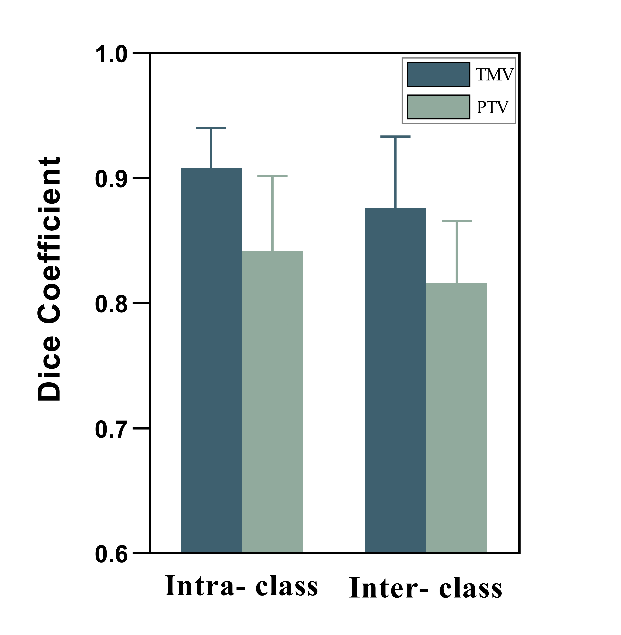
**

**Figure 1** The intra-class Dice coefficient of TMV was 0.908±0.032, and the inter- was 0.876±0.057; the intra-class Dice coefficient of PTV was 0.842±0.060, and the inter- was 0.816±0.050

**Figure 2**


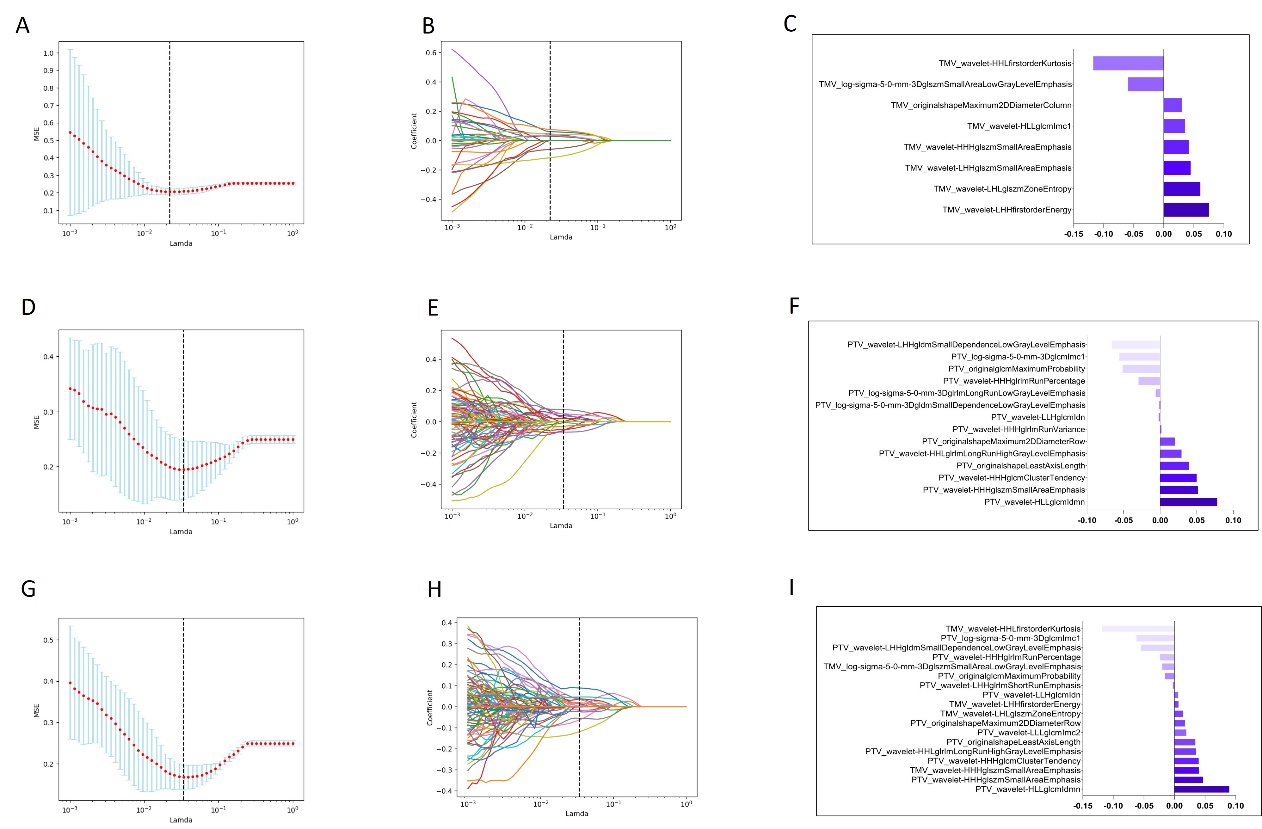


**Figure 2** (A,B,C):TMV model ;(D,E,F):PTV model ;(G,H,I):TM-PTV model. Use of lasso logistic regression to select features. (A, D, G)：LASSO regression is used to screen radiomics features. The vertical axis represents the mean absolute error, the horizontal axis represents the logarithmic value(λ), and the vertical dashed line represents the best binomial deviation of the model. (B, E, H): Variation of non-zero eigencoefficients with λ.(C, F, I): the coefficients of the filtered features.

**
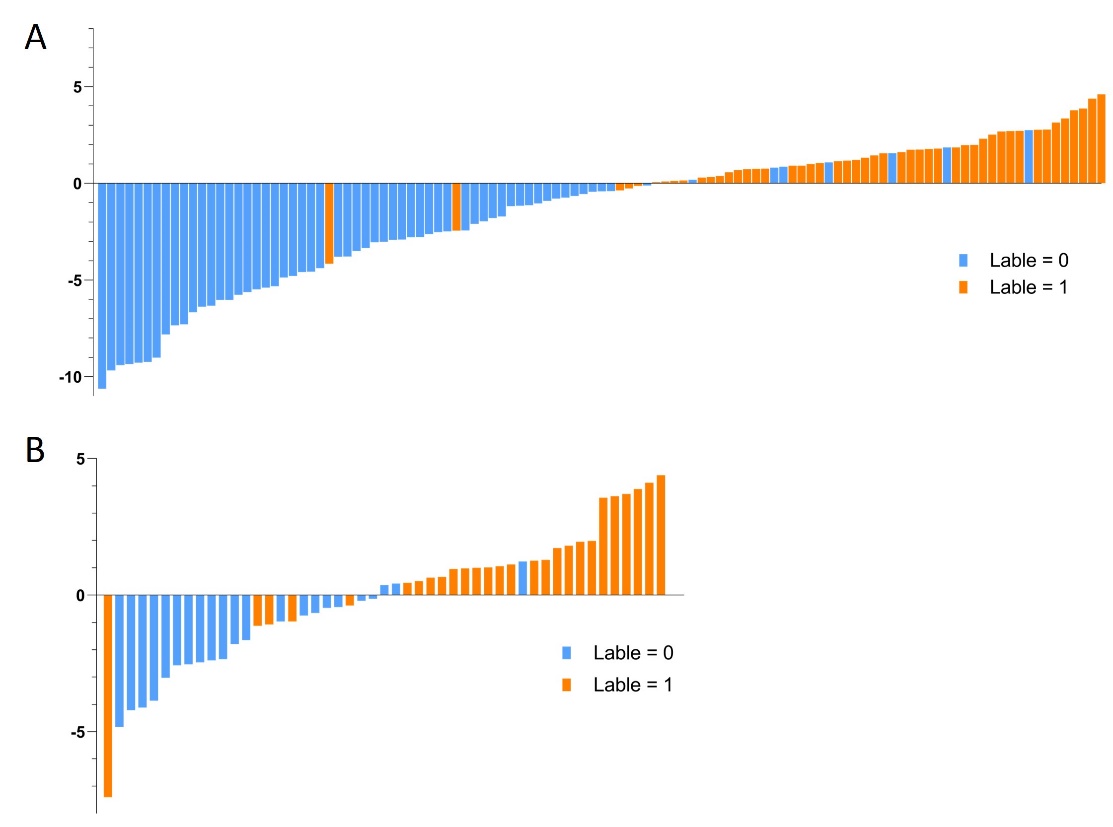
**

**Figure 3.** Rad-score for every patient in each cohort. (A) Rad-score for every patient in the training cohort; (B) Rad-score for every patient in the validation cohort. The status of the grade was marked with different colors.
